# Use of the atmospheric generators for capnophilic bacteria Genbag-CO2 for the evaluation of in vitro *Plasmodium falciparum *susceptibility to standard anti-malarial drugs

**DOI:** 10.1186/1475-2875-10-8

**Published:** 2011-01-14

**Authors:** Aurélie Pascual, Leonardo K Basco, Eric Baret, Rémy Amalvict, Dominique Travers, Christophe Rogier, Bruno Pradines

**Affiliations:** 1 Unité de Recherche en Biologie et Epidémiologie Parasitaires, Institut de Recherche Biomédicale des Armées - antenne de Marseille, Marseille, France; 2 Unité de Recherche sur les Maladies Infectieuses et Tropicales Emergentes - UMR 6236, Marseille, France; 3 Centre national de référence du paludisme, Marseille, France

## Abstract

**Background:**

The aim of this study was to evaluate the cultivation system in which the proper atmospheric conditions for growing *Plasmodium falciparum *parasites were maintained in a sealed bag. The Genbag^® ^system associated with the atmospheric generators for capnophilic bacteria Genbag CO2^® ^was used for *in vitro *susceptibility test of nine standard anti-malarial drugs and compared to standard incubator conditions.

**Methods:**

The susceptibility of 36 pre-identified parasite strains from a wide panel of countries was assessed for nine standard anti-malarial drugs (chloroquine, quinine, mefloquine, monodesethylamodiaquine, lumefantrine, dihydroartemisinin, atovaquone and pyrimethamine) by the standard 42-hour ^3^H-hypoxanthine uptake inhibition method using the Genbag CO2^® ^system and compared to controlled incubator conditions (5% CO_2 _and 10% O_2_).

**Results:**

The counts per minute values in the control wells in incubator atmospheric conditions (5% CO_2 _and 10% O_2_) were significantly higher than those of Genbag^® ^conditions (2738 cpm vs 2282 cpm, p < 0.0001). The geometric mean IC_50 _estimated under the incubator atmospheric conditions was significantly lower for atovaquone (1.2 vs 2.1 nM, p = 0.0011) and higher for the quinolines: chloroquine (127 vs 94 nM, p < 0.0001), quinine (580 vs 439 nM, p < 0.0001), monodesethylamodiaquine (41.4 vs 31.8 nM, p < 0.0001), mefloquine (57.5 vs 49.7 nM, p = 0.0011) and lumefantrine (23.8 vs 21.2 nM, p = 0.0044). There was no significant difference of IC_50 _between the 2 conditions for dihydroartemisinin, doxycycline and pyrimethamine.

To reduce this difference in term of anti-malarial susceptibility, a specific cut-off was estimated for each drug under Genbag^® ^conditions by regression. The cut-off was estimated at 77 nM for chloroquine (vs 100 nM in 10% O_2_), 611 nM for quinine (vs 800 nM), 30 nM for mefloquine (vs 30 nM), 61 nM for monodesethylamodiaquine (vs 80 nM) and 1729 nM for pyrimethamine (vs 2000 nM).

**Conclusions:**

The atmospheric generators for capnophilic bacteria Genbag CO2^® ^is an appropriate technology that can be transferred to the field for epidemiological surveys of drug-resistant malaria. The present data suggest the importance of the gas mixture on *in vitro *microtest results for anti-malarial drugs and the importance of determining the microtest conditions before comparing and analysing the data from different laboratories and concluding on malaria resistance.

## Background

Over the past 20 years, many strains of *Plasmodium falciparum *have become resistant to chloroquine and other anti-malarial drugs [1]. Since 2001, more than 60 countries have officially adopted artemisinin-based combination therapies (ACTs) for the treatment of falciparum malaria [2,3]. However, clinical failures or at least longer parasite clearance times with ACT have been described in Cambodia [4-7]. The emergence and spread of resistance to most of the anti-malarial drugs require intensive research into identifying molecular markers of resistance, as well as implementing *in vitro *and *in vivo *surveillance programmes, such as those supported by the Worldwide Antimalarial Resistance Network [8,9].

There are basically three approaches to assess anti-malarial drug susceptibility of *P. falciparum*: assessment of therapeutic efficacy standardized by the World Health Organization (WHO) [10], *in vitro *assays and molecular markers of resistance.

In a number of laboratories surveying anti-malarial drug resistance, *in vitro *tests are performed using the uptake of a radiolabelled nucleic acid precursor [^3^H]-hypoxanthine as a marker of parasite growth [11]. Other non-radioactive methods can be used: the WHO schizont maturation test by optical microscopy (Mark III) with pre-dosed plates [12], which was based on the methods of Rieckmann *et al *[13] and Wernsdorfer [14], a flow cytometric analysis of propidium iodide incorporation into parasite, which permits a stage-specific evaluation of anti-malarial compounds [15], a fluorescent-based technique that uses SYBR green I which binds to DNA [16,17], and colorimetric or enzyme-linked immunosorbent assays (ELISA) to measure histidine-rich protein II (HRP2) [18,19] or plasmodial lactate dehydrogenase enzyme (pLDH) [20,21].

Many factors induce high variations in *P. falciparum *growth and 50% inhibitory concentration (IC_50_) values and influence the results of the chemosusceptibility tests [22], such as culture medium, initial parasitaemia, haematocrit, incubation time, time point when [^3^H]-hypoxanthine is added, use of serum substitutes, storage conditions of sample, delay before cultivation of samples and atmosphere (gas mixtures).

Laboratories using isotopic microtest to monitor drug resistance work at different oxygen tensions: 3% O_2 _[15], 5% O_2 _[18,19], 10% O_2 _[23,24], in candle jars [20,25] (which corresponds to approximately 17-18% O_2_) and >20% O_2 _[21,22] (in CO_2 _incubators). WHO recommends the use of a candle jar in their *in vitro *microtests (Mark III). Despite the varying culture conditions, many laboratories have adopted the same threshold for the resistance to anti-malarial compounds under different oxygen tensions. For example, our previous study has shown that the chloroquine IC_50 _values at 10% O_2 _were significantly higher than those at 21% O_2 _[26]. Nevertheless, it seems that O_2 _concentrations between 1% and 17.5% do not affect the IC_50 _values of quinoline-containing anti-malarial drugs [27-29]. In contrast, the *in vitro *anti-malarial activity of some antibiotics was dependent on the O_2 _concentration [27].

The aim of this study was to evaluate a cultivation system in which the proper conditions of atmosphere for growing *P. falciparum *parasites were maintained in an air-tight sealed bag. The Genbag^® ^system was initially designed as atmospheric generators for capnophilic bacteria. Genbag CO2^® ^(BioMérieux;Marcy l'Etoile, France) was used for *in vitro *susceptibility test of nine standard anti-malarial drugs, and the IC_50_s were compared to those obtained with controlled incubator conditions (5% CO_2_, 10% O_2 _and 85% N_2_).

## Methods

### Strains of *P. falciparum*

A total of 36 pre-identified parasite strains (well-characterized laboratory strains or strains obtained from isolates after growth in culture for an extended period of time) from a wide panel of countries were maintained in culture in RPMI 1640 (Invitrogen, Paisley, United Kingdom), supplemented with 10% human serum (Abcys S.A., Paris, France) and buffered with 25 mM HEPES and 25 mM NaHCO_3_. Parasites were grown in type A^+ ^human red blood cells under controlled atmospheric conditions that consisted of 10% O_2_, 5% CO_2 _and 85% N_2 _at 37°C with a relative humidity of 95%. All strains were synchronized twice with sorbitol before use [30]. Clonality was verified using PCR genotyping of polymorphic genetic markers *msp1*, *msp2*, and microsatellite loci [31,32]. Chloroquine-susceptible 3D7 clone and chloroquine-resistant W2 clone (MR4 Resource Center) were cultivated in the same conditions and assessed for drug susceptibility in 4 independent experiments.

### Drugs

Chloroquine, quinine, dihydroartemisinin, pyrimethamine and doxycycline were purchased from Sigma (Saint Louis, MO). Monodesethylamodiaquine was obtained from the WHO (Geneva, Switzerland). Mefloquine was obtained from Roche (Paris, France). Lumefantrine was provided by Novartis Pharma (Basel, Switzerland), and atovaquone was from GlaxoSmithKline (Evreux, France). Chloroquine and pyrimethamine were dissolved and diluted in water to obtain final concentrations ranging from 5 to 3,200 nM for chloroquine and 5 to 40,000 nM for pyrimethamine. Quinine, monodesethylamodiaquine, mefloquine, dihydroartemisinin, atovaquone and doxycycline were first dissolved in methanol and then diluted in water to obtain final concentrations ranging from 5 to 3,200 nM for quinine, 1.56 to 1000 nM for monodesethylamodiaquine, 3.2 to 400 nM for mefloquine, 0.1 to 100 nM for dihydroartemisinin, 0.3 to 100 nM for atovaquone and 0.1 to 502 μM for doxycycline. Lumefantrine was dissolved and diluted in ethanol to obtain final concentrations ranging from 0.5 to 310 nM.

### In vitro assay

**Figure 1:**
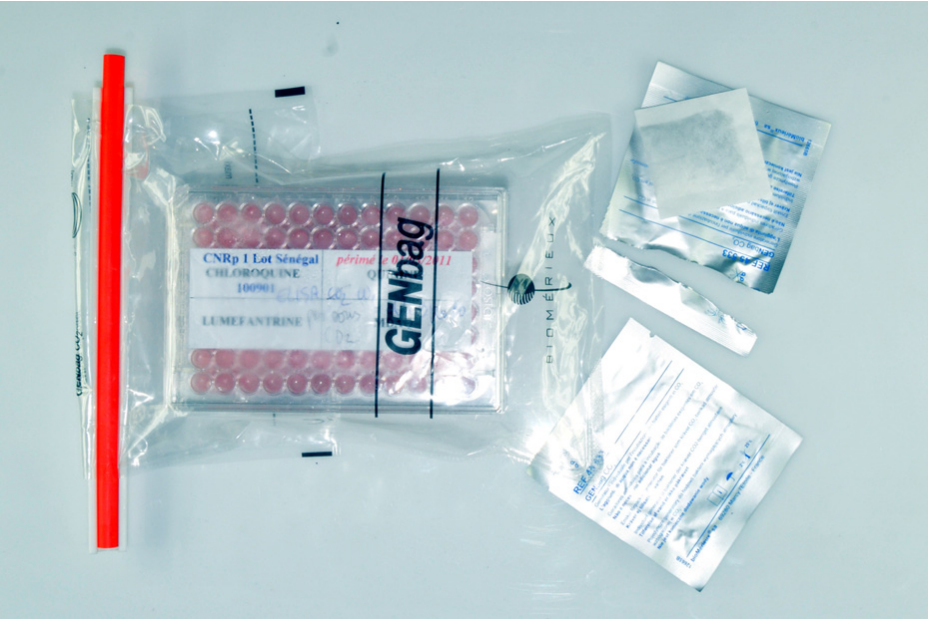
**The Genbag^® ^system associated with the atmospheric generators for capnophilic bacteria Genbag CO2^® ^with 2 plates in the specific sealed bag (picture P Millileri)**.

For *in vitro *isotopic microtests, 200 μL/well of a suspension of synchronous parasitized red blood cells (final parasitaemia, 0.5%; final haematocrit, 1.5%) was distributed in 96-well plates predosed with anti-malarial drugs. Parasite growth was assessed by adding 1 μCi of tritiated hypoxanthine with a specific activity of 14.1 Ci/mmol (Perkin-Elmer, Courtaboeuf, France) to each well at time zero. The plates were incubated for 42 h at 37°C in controlled atmospheric conditions in incubator (5% CO_2_, 10% O_2 _and 85% N_2_) and Genbag^® ^with the atmospheric generators for capnophilic bacteria Genbag CO2^® ^(two plates per sealed bag) (Figure 1). Immediately after incubation, plates were frozen and then thawed to lyse the erythrocytes. The contents of each well were collected on standard filter microplates (Unifilter GF/B; Perkin-Elmer) and washed using a cell harvester (Filter-Mate Cell Harvester; Perkin-Elmer). The filter microplates were dried, and 25 μL of scintillation cocktail (Microscint O; Perkin-Elmer) were placed in each well. The radioactivity incorporated into the nucleotides of the parasites was measured with a scintillation counter (Top Count; Perkin-Elmer).

The drug concentration that inhibits 50% of parasite growth (IC_50_) was designated as the concentration at which the tritiated hypoxanthine incorporation reached 50% of the total incorporation by the parasites in the drug-free control wells. The IC_50 _value was determined by non-linear regression analysis of log-based dose-response curves (Riasmart, Packard, Meriden, USA).

### Statistical analysis

Count per minute (cpm) and IC_50 _were expressed as geometric means and 95% confidence intervals. The differences between the data observed in the incubator and Genbag^® ^were analysed by a paired *t*-test. The correlation between the responses under two incubation conditions for each anti-malarial drug was estimated by the Pearson correlation coefficient R. The same analyses were performed on IC_50 _strains/IC_50 _3D7 or IC_50 _strains/IC_50 _W2 ratios calculated for each anti-malarial drug under the two conditions.

## Results

A total of 1,098 drug-free control wells were analysed for each experimental condition. A significant difference was observed in the tritiated hypoxanthine uptake between the two conditions with 36 strains of *P. falciparum *(Table 1). The mean cpm values in the control wells under the controlled incubator conditions (5% CO_2 _and 10% O_2_) were significantly higher than those under Genbag^® ^conditions (2738 vs 2282 cpm, p value < 0.0001).

**Table 1:** In vitro activity of chloroquine, quinine, mefloquine, monodesethylamodiaquine, lumefantrine, pyrimethamine, atovaquone, dihydroartemisinin and doxycycline against 36 strains of *Plasmodium falciparum *under controlled incubator conditions (10% O_2_) and Genbag^® ^conditions (15% O_2_)

Compound	**O**_**2 **_**%**	**IC**_**50**_				t-test p-value
			
		Geometric mean	95%CI	Min	Max	
Chloroquine	10	127 nM	77-211	8	1032	<0.0001
	15	94 nM	61-146	12	751	
Quinine	10	580 nM	451-745	153	1752	<0.0001
	15	439 nM	336-573	96	1288	
Mefloquine	10	57.5 nM	50.7-65.3	30.8	131.0	0.0011
	15	49.7 nM	44.0-46.3	26.4	113.0	
Monodesethylamodiaquine	10	41.4 nM	28.8-59.6	4.7	379	<0.0001
	15	31.8 nM	21.8646.2	4.0	357	
Lumefantrine	10	23.8 nM	19.6628.8	7.1	65.0	0.0044
	15	21.2 nM	17.5-25.7	7.2	72	
Pyrimethamine	10	273 nM	88-852	10	17025	0.0902
	15	222 nM	67-735	10	14938	
Atovaquone	10	1.2 nM	0.8-1.8	0.1	7.3	0.0011
	15	2.1 nM	1.6-2.8	0.2	11.7	
Dihydroartemisinin	10	2.4 nM	2.0-2.9	1.2	10.8	0.4088
	15	2.4 nM	1.9-2.9	1.0	14.3	
Doxycycline	10	10.9 μM	9.8-12.1	7.6	26.0	0.6771
	15	10.8 μM	9.5-12.2	6.3	24.5	
cpm	10	2738	2628-2852	671	17845	<0.0001
	15	2282	2189-2379	623	12726	

Compared to Genbag^® ^conditions (5% CO_2 _and 15% O_2_), the geometric mean IC_50 _estimated under the incubator atmospheric conditions (5% CO_2 _and 10% O_2_) was significantly lower for atovaquone (1.2 vs 2.1 nM, p = 0.0011) and higher for the quinolines: chloroquine (127 vs 94 nM, p < 0.0001), quinine (580 vs 439 nM, p < 0.0001), monodesethylamodiaquine (41.4 vs 31.8 nM, p < 0.0001), mefloquine (57.5 vs 49.7 nM, p = 0.0011) and lumefantrine (23.8 vs 21.2 nM, p = 0.0044) (Table 1). There was no significant difference of IC_50 _between the two conditions for dihydroartemisinin, doxycycline and pyrimethamine.

**Figure 2:**
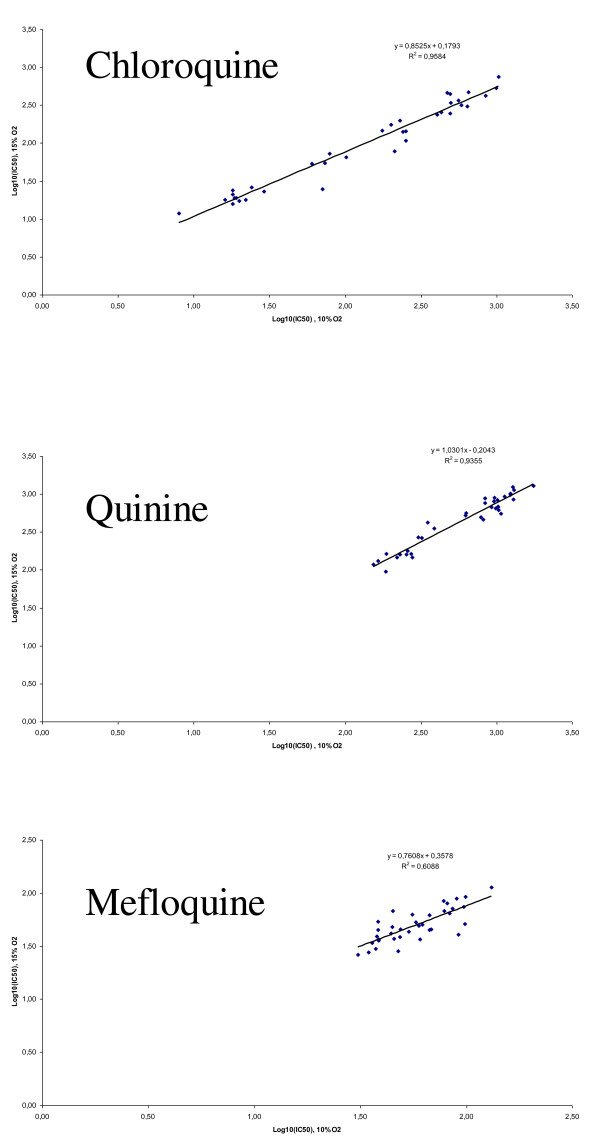
**Correlation between log IC**_**50 **_**values of chloroquine, quinine or mefloquine, against 36 strains of *Plasmodium falciparum *under controlled incubator conditions (10% O**_**2**_**) and Genbag^® ^conditions (15% O**_**2**_**)**.

**Figure 3:**
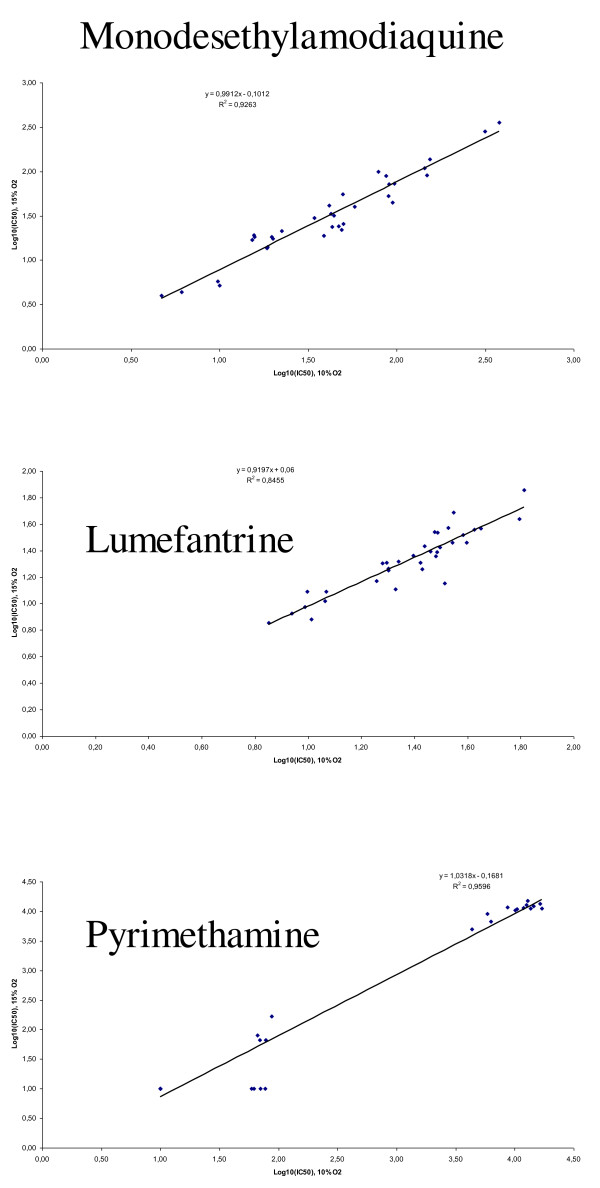
**Correlation between log IC**_**50 **_**values of monodesethylamodiaquine, lumefantrine or pyrimethamine against 36 strains of *Plasmodium falciparum *under controlled incubator conditions (10% O**_**2**_**) and Genbag^® ^conditions (15% O**_**2**_**)**.

**Figure 4:**
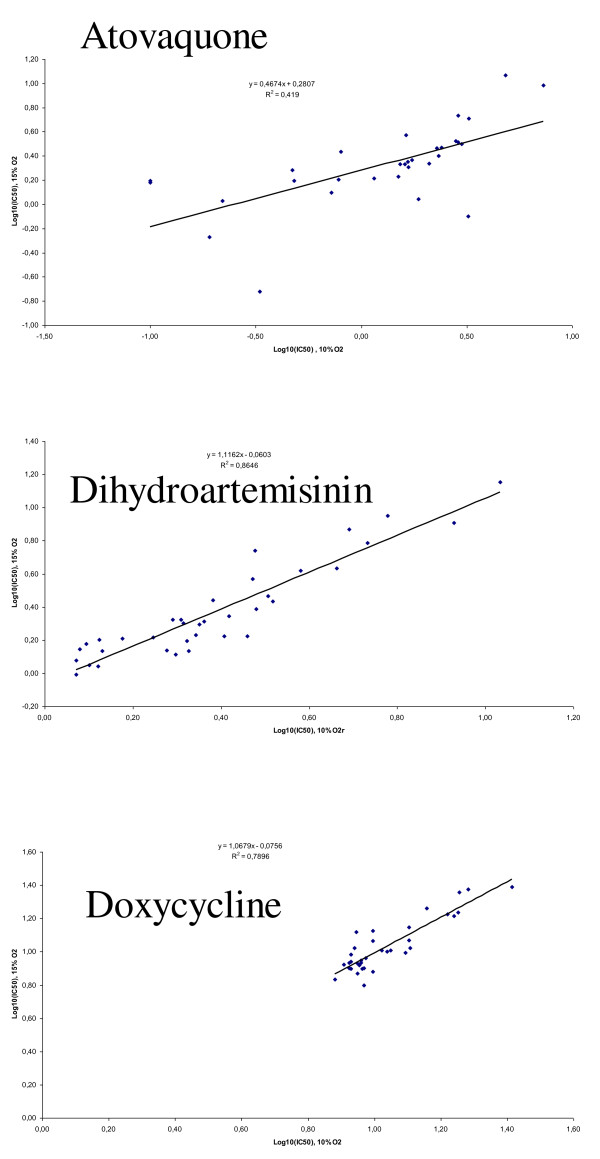
**Correlation between log IC**_**50 **_**values of atovaquone, dihydroartemisinin or doxycycline against 36 strains of *Plasmodium falciparum *under controlled incubator conditions (10% O**_**2**_**) and Genbag^® ^conditions (15% O**_**2**_**)**.

The cpm and IC_50 _values for each anti-malarial drug were highly and significantly correlated between the two conditions (Figure 2, 3 and 4).

Based on the thresholds of decreased susceptibility defined for the incubator atmospheric conditions (5% CO_2 _and 10% O_2_), the following discrepancies were observed: one of 21 strains (5%) with IC_50 _>100 nM for chloroquine [33] was <100 nM under Genbag^® ^conditions; seven of 20 strains (35%) with IC_50 _>800 nM for quinine [34] was <800 nM under Genbag^® ^conditions; two of 20 strains (10%) with IC_50 _>30 nM for mefloquine [35] was <30 nM under Genbag^® ^conditions; four of 10 strains (40%) with IC_50 _>80 nM for monodesethylamodiaquine [36] was <80 nM under Genbag^® ^conditions; and none of the 13 strains with IC_50 _>2,000 nM for pyrimethamine [37] was < 2,000 nM under Genbag^® ^conditions.

To reduce this difference in terms of anti-malarial susceptibility, a specific cut-off was estimated for each drug under Genbag^® ^conditions by regression. The cut-off was estimated at 77 nM for chloroquine (vs 100 nM in 10% O_2_), 611 nM for quinine (vs 800 nM), 30 nM for mefloquine (vs 30 nM), 61 nM for monodesethylamodiaquine (vs 80 nM) and 1729 nM for pyrimethamine (vs 2,000 nM). The cut-off was not re-estimated for drugs for which all the CI_50 _values were below the cut-off under controlled incubation conditions. For these drugs, median values were estimated: 2.9 nM for atovaquone (vs 2.5 nM in 10% O_2_), 26 nM for lumefantrine (vs 30 nM), 3 nM for dihydroartemisinin (vs 3.0 nM) and 15 μM for doxycycline (vs 15 μM).

The geometric mean ratio based on the chloroquine-susceptible reference clone 3D7 (strain IC_50_/3D7 IC_50_) obtained under the incubator atmospheric conditions (5% CO_2 _and 10% O_2_) was significantly higher for chloroquine (p < 0.0001), lumefantrine (p = 0.0119), and dihydroartemisinin (p = 0.0006), but the ratio was significantly lower for mefloquine (p < 0.0001) and doxycycline (p = 0.0002) (Table 2). The geometric mean ratio based on the chloroquine-resistant reference clone W2 (strain IC_50_/W2 IC_50_) obtained under the incubator atmospheric conditions (5% CO_2 _and 10% O_2_) was significantly higher for chloroquine (p = 0.0014), quinine (p = 0.0006), monodesethylamodiaquine (p < 0.0001), lumefantrine (p < 0.0001), pyrimethamine (p = 0.0026) and was significantly lower for dihydroartemisinin (p < 0.0001) and doxycycline (p = 0.0159) (Table 3).

**Table 2:** Ratio based on the chloroquine-susceptible reference clone 3D7 (strain IC_50_/3D7 IC_50_) of chloroquine, quinine, mefloquine, monodesethylamodiaquine, lumefantrine, pyrimethamine, atovaquone, dihydroartemisinin and doxycycline against 36 strains of *Plasmodium falciparum *under controlled incubator conditions (10% O_2_) and Genbag^® ^conditions (15% O_2_)

Compound	**O**_**2 **_**%**	**IC**_**50 **_**/3D7 IC**_**50**_				t-test p-value
			
		Geometric mean	95%CI	Min	Max	
Chloroquine	10	6.88	4.15-11.41	0.40	55.80	<0.0001
	15	4.80	3.09-7.45	0.60	38.30	
Quinine	10	2.44	1.90-3.13	0.65	7.39	0.2418
	15	2.63	2.04-3.40	0.66	7.24	
Mefloquine	10	0.72	0.63-0.82	0.39	1.64	<0.0001
	15	0.91	0.81-1.03	0.49	2.089	
Monodesethylamodiaquine	10	2.59	1.80-3.72	0.29	23.69	0.5948
	15	2.52	1.73-3.67	0.32	28.33	
Lumefantrine	10	0.90	0.74-1.09	0.27	2.45	0.0119
	15	0.81	0.67-0.98	0.28	2.76	
Pyrimethamine	10	27.31	8.75-85.21	1.00	1703	0.0835
	15	18.01	5.51-58.82	1.00	1494	
Atovaquone	10	0.88	0.59-1.31	0.07	5.20	0.9187
	15	0.89	0.67-1.19	0.08	4.96	
Dihydroartemisinin	10	1.53	1.27-1.85	0.74	6.79	0.0006
	15	1.31	1.04-1.64	0.54	7.94	
Doxycycline	10	1.21	1.09-1.35	0.85	2.90	0.0002
	15	1.37	1.21-1.55	0.80	3.11	

**Table 3:** Ratio based on the chloroquine-resistant reference clone W2 (strain IC_50_/W2 IC_50_) of chloroquine, quinine, mefloquine, monodesethylamodiaquine, lumefantrine, pyrimethamine, atovaquone, dihydroartemisinin and doxycycline against 36 strains of *Plasmodium falciparum *under controlled incubator conditions (10% O_2_) and Genbag^® ^conditions (15% O_2_)

Compound	**O**_**2 **_**%**	**IC**_**50 **_**/W2 IC**_**50**_				t-test p-value
			
		Geometric mean	95%CI	Min	Max	
Chloroquine	10	0.26	0.16-0.43	0.02	2.12	0.0014
	15	0.21	0.14-0.33	0.03	1.71	
Quinine	10	0.71	0.56-0.90	0.19	2.04	0.0006
	15	0.56	0.43-0.74	0.12	1.65	
Mefloquine	10	0.97	0.66-1.42	0.43	1.82	0.6262
	15	0.82	0.72-0.92	0.43	1.85	
Monodesethylamodiaquine	10	0.49	0.34-0.70	0.06	4.47	<0.0001
	15	0.37	0.25-0.54	0.05	4.14	
Lumefantrine	10	1.25	1.03-1.52	0.37	3.42	<0.0001
	15	0.94	0.78-1.15	0.32	3.21	
Pyrimethamine	10	0.03	0.01-0.10	0.001	1.96	0.0026
	15	0.02	0.01-0.07	0.0008	1.26	
Atovaquone	10	0.65	0.44-0.97	0.05	3.85	0.8646
	15	0.67	0.50-0.89	0.06	3.71	
Dihydroartemisinin	10	1.06	0.88-1.28	0.51	4.70	<0.0001
	15	1.55	1.24-1.94	0.65	9.41	
Doxycycline	10	1.16	1.05-1.29	0.81	2.78	0.0159
	15	1.25	11.10-1.42	0.73	2.85	

## Discussion

The first works that assessed oxygen effects on *P. falciparum *asynchronous cultures had shown that microaerophilic environment allowed an optimal development of parasites [38]. Parasite growth failed under strict anaerobic conditions. *Plasmodium falciparum *possesses a functional mitochondrial respiratory chain with oxygen consumption [39]. It has been shown that there is some protector effect of CO_2 _at high oxygen concentration [38] through the medium pH, the stability (between 7.2 and 7.45) of which is required for parasite growth [40]. The standard medium RPMI 1640, buffered with 25 mM HEPES and 25 mM NaHCO_3_, was optimized to maintain the pH within the physiological range in an atmosphere containing 5% CO_2_. Any modification of the CO_2 _concentration alters the pH of the medium, which in turn can influence the IC_50 _values of pH-dependent drugs, such as quinolines, but not of those that are pH-independent, such as pyrimethamine. Despite similar growth and tritium-labelled hypoxanthine incorporation rates in drug-free control wells, Shenyi He *et al *showed that increasing the CO_2 _concentration from 2.7% to 7% (with a constant 5% O_2_) resulted in significantly higher chloroquine IC_50 _values [29]. The chloroquine-resistant K1 strain showed nine-fold greater chloroquine IC_50 _when the CO_2 _concentration was increased from 2.7 to 7% [29].

The atmospheric generators for capnophilic bacteria Genbag CO_2_^® ^evaluated in this study release about 5% CO_2 _and reduce to 15% O_2 _in 30 min, according to the manufacturer's specifications (http://www.biomerieux-diagnostics.com/servlet/srt/bio/clinical-diagnostics/dynPage?doc=CNL_PRD_CPL_G_PRD_CLN_62). The stated conditions are maintained at least for 48 h. In this context, it seems that the significant differences in drug IC_50 _values and growth level (cpm) between the two conditions, the incubator atmospheric conditions (5% CO_2 _and 10% O_2_) and the Genbag^® ^conditions (5% CO_2 _and 15% O_2_), are due to the difference in O_2 _concentration.

The IC_50 _values of quinoline drugs, such as chloroquine, quinine, monodesethylamodiaquine, mefloquine and lumefantrine were significantly lower at 15% O_2 _(Genbag CO_2_^® ^) than those at 10% O_2 _(the incubator atmospheric conditions). This is in agreement with the previous results that showed in 136 *P. falciparum *fresh isolates from Comoros that the chloroquine IC_50 _values at 10% O_2 _were significantly higher than those at 21% O_2_, with the means of 173.5 nM and 121.5 nM, respectively [26]. Of particular interest among the 63 isolates that were resistant *in vitro *to chloroquine (IC_50 _>100 nM) at 5% CO_2 _and 10% O_2_, was the observation that 17 were susceptible to chloroquine (IC_50 _< 100 nM) at 21% O_2 _[26]. In the present study, only one of 21 strains with chloroquine IC_50 _>100 nM at 10% O_2 _had IC_50 _< 100 nM at a concentration of 15%. Some studies failed to show oxygen-dependent effects of chloroquine on *P. falciparum *in culture [27-29], but in these experiments less than four strains were tested. Shenyi He *et al *reported that the chloroquine IC_50 _values determined in a candle jar (2.7% CO_2 _and 17.5% O_2_) were similar to those determined in an incubator set at 2.7% CO_2 _and 5% O_2 _[27]. Lin *et al *observed similar chloroquine IC_50 _values with parasites incubated in 5% CO_2 _and 5% or 15% O_2 _generated by an AnaeroPack system [29].

In contrast, there was no significant difference of IC_50 _values between 10% and 15% O_2 _conditions for doxycycline, dihydroartemisinin and pyrimethamine. Divo *et al*. reported that the anti-malarial activity of different cyclines was O_2_-dependent and higher at high O_2 _concentrations [27]. Nevertheless, this influence of O_2 _was not evident at 48 h but was profound at 96 h. In the present study, IC_50 _values were only determined at 42 h. The class of artemisinins contains an intramolecular peroxide bridge that is situated in the sesquiterperne lactone backbone structure. The anti-malarial potency of artemisinin was enhanced by oxygen and inhibited by oxygen radical scavengers [41,42]. The contrast between 10 and 15% O_2 _is probably not high enough to observe any difference in dihydroartemisinin IC_50 _values.

Using the threshold values for in vitro resistance established under the controlled incubator conditions (5% CO_2 _and 10% O_2_), 0 - 40% of discordant results, depending on the test compounds, was obtained using the Genbag^® ^incubation system. The effect of gas mixture on the results of chemosusceptibility assay should lead different laboratories involved in anti-malarial resistance survey to adapt a resistance threshold for each gas mixture or to use the same conditions to perform chemosusceptibility microtests. The cut-off value was re-estimated for each drug in Genbag^® ^conditions. The cut-off was estimated at 77 nM for chloroquine (vs 100 nM in 10% O_2_), 611 nM for quinine (vs 800 nM), 30 nM for mefloquine (vs 30 nM), 61 nM for monodesethylamodiaquine (vs 80 nM) and 1729 nM for pyrimethamine (vs 2000 nM).

To reduce the effects of O_2 _on IC_50 _values between 10 and 15% O_2_, the ratios of strain IC_50_/3D7 IC_50 _and strain IC_50_/W2 IC_50 _were calculated for each condition. Nevertheless, the mean ratios were not similar at 10% and 15% O_2 _for most anti-malarial drugs, and the results depended on the reference clone.

## Conclusions

The atmospheric generators for capnophilic bacteria Genbag CO2^® ^is an appropriate technology that can be transferred to the field for epidemiological surveys of drug-resistant malaria. The present data suggest the importance of the gas mixture on *in vitro *microtest results for anti-malarial drugs and the importance of determining the microtest conditions before comparing and analysing the data from different laboratories and concluding on malaria resistance.

## Conflict of interest

The authors declare that they have no competing interests.

## Authors' contributions

LKB and BP conceived and designed the experiments. AP, EB, RA and DT performed the *in vitro *experiments. CR and BP analysed the data. AP, LKB, CR and BP wrote the paper. All authors read and approved the final manuscript.
